# Ganglioside GD1a enhances osteogenesis by activating ERK1/2 in mesenchymal stem cells of *Lmna* mutant mice

**DOI:** 10.18632/aging.204378

**Published:** 2022-11-14

**Authors:** Dong Hoon Kwak, Ji Hye Park, Eul Sig Choi, Seong Hyun Park, Seo-Yeon Lee, Seoul Lee

**Affiliations:** 1 Department of Pharmacology, School of Medicine, Wonkwang University, Iksan, Jeollabuk-do 54538, Republic of Korea; 2 Brain Research Institute, Wonkwang University, Iksan, Jeollabuk-do 54538, Republic of Korea

**Keywords:** Lmna, mesenchymal stem cells, osteogenesis, GD1a, aging

## Abstract

Mutations in *Lmna* usually cause a series of human disorders, such as premature aging syndrome (progeria) involving the skeletal system. Gangliosides are known to be involved in cell surface differentiation and proliferation of stem cells. However, the role of gangliosides in *Lmna* dysfunctional mesenchymal stem cells (MSCs) is unclear. Therefore, Ganglioside's role in osteogenesis of Lmna dysfunctional MSCs analyzed. As a result of the analysis, it was confirmed that the expression of ganglioside GD1a was significantly reduced in MSCs derived from *Lmna*^Dhe/+^ mice and in MSCs subjected to Lamin A/C knockdown using siRNA. Osteogenesis-related bone morphogenetic protein-2 and Osteocalcin protein, and gene expression were significantly decreased due to *Lmna* dysfunction. A result of treating MSCs with *Lmna* dysfunction with ganglioside GD1a (3 μg/ml), significantly increased bone differentiation in ganglioside GD1a treatment to *Lmna*-mutated MSCs. In addition, the level of pERK1/2, related to bone differentiation mechanisms was significantly increased. Ganglioside GD1a was treated to Congenital progeria *Lmna*^Dhe/+^ mice. As a result, femur bone volume in ganglioside GD1a-treated *Lmna*^Dhe/+^ mice was more significantly increased than in the *Lmna*^Dhe/+^ mice. Therefore, it was confirmed that the ganglioside GD1a plays an important role in enhancing osteogenic differentiation in MSC was a dysfunction of *Lmna*.

## INTRODUCTION

The *Lmna* gene encodes Lamin A/C. An essential scaffolding component of the nuclear membrane that surrounds the cell nucleus is the *Lmna*. Lamin A/C plays a role in chromatin structure, nuclear stability, and gene expression [1]. Interest in the *Lmna* gene-associated proteins has increased as many mutations in the *Lmna* gene have been shown to cause a wide range of connective tissue, neuropathology, and premature aging syndromes (progeria) in human patients [2]. The term “laminopathy” was coined to describe these genetic disorders, the term is extended to “nuclear enteropathies” to include similar syndromes caused by mutations in other proteins in the nuclear membrane [3].

Mutations in *Lmna* commonly cause a series of human disorders. Phenotypes associated with mutations in human *Lmna* include defects in skin, cardiac and adipocytes, peripheral nerves, skeletal, and other tissues. Skeletal involvement is reported in Progeria, in which the bone is hypoplasia, the clavicle is dysplastic, and the joint is defective [4]. The *Lmna* mutation, first described by Odgren et al. [5], was named for an external phenotype of sparse hair and small ears, called disheveled hair and ears (*Dhe*). Mutations have also been related to craniofacial defects and epidermal dysplasia in both heterozygotes and homozygotes. However, homozygotes rarely survive after P10.8.

Numerous studies have confirmed that gangliosides and their expression levels are regulated during development [6] and are cell type dependent [7]. Some biological roles of ganglioside include cell proliferation [8], cell adhesion [9], apoptosis [10], and differentiation [11] in stem cells. Also, a decrease in ganglioside biosynthesis has been shown to inhibit the neuronal differentiation of MSCs at an early stage in the process [12], and recently demonstrated that an increase in the ganglioside GD1a is important in differentiation of human MSCs [13].

We hypothesize that ganglioside has an important relationship in the *Lmna* mutation model, but the distribution and role of ganglioside in the *Lmna* mutation model are unclear. Bone formation by Lmna mutation appears to be affected, but to date, it has not been sufficiently investigated to confirm this definitively. There are no studies on bone formation and gangliosides in mesenchymal stem cells due to loss of Lamin A function due to *Lmna* mutation. Therefore, in this study, the distribution and role of ganglioside in bone formation of *Lmna^Dhe/+^* mutant MSC, an *Lmna* mutation model, was investigated.

## RESULTS

### Freshly isolated mesenchymal stem cells from mouse bone marrow

We isolated MSCs from the bone marrow of *Lmna^Dhe/+^* mutant and normal mice. To identify fresh MSCs, we performed FACS analysis using antibodies CD73 and CD90, which are positive markers of MSCs, and antibodies, CD34 and CD45, which are negative markers. As a result of the analysis, CD73 and CD90 which are general markers of MSCs were positive, and CD34 and CD45 were negative (Supplementary Figure 1). These results suggest that cells isolated from mice are fresh MSCs. Expression of Markers of *Lmna^Dhe/+^* mutant MSCs and normal MSCs is similar.

### Micro-array analysis of genes expression in *Lmna^Dhe/+^* mutant MSCs compared to normal MSCs

**Figure 1:**
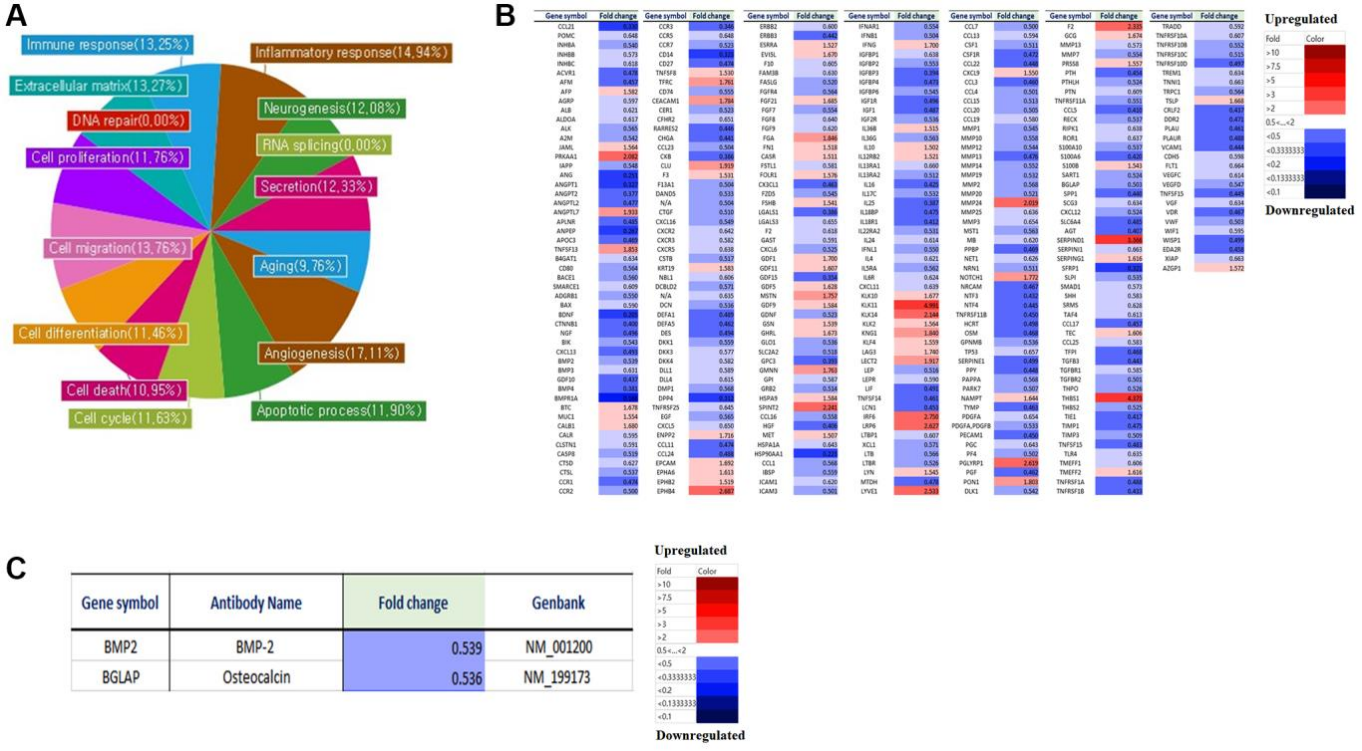
**Micro-array analysis of genes expression in *Lmna^Dhe/+^* mutant MSCs compared to normal MSCs.** Gene expression was analyzed by a microarray using an antibody. (**A**) Antibody microarray assay (Ray L1000 Antibody Array) can analyze 1000 genes and has 15 domains such as inflammatory response, neurogenesis, RNA splicing, secretion, extracellular matrix, angiogenesis, cell cycle, apoptotic processes, cell differentiation, cell death, cell migration, cell proliferation, DNA repair, aging, and immune response. (**B**) We analyzed the differences in gene expression in *Lmna^Dhe/+^ mutant MSC* compared to normal MSC. (**C**) Decreases of osteogenesis-related genes expression in *Lmna^Dhe/+^* mutant MSCs compared to normal MSCs.

We analyzed the differences in gene expression in LmnaDhe/+ mutant MSCs compared to normal MSCs. Gene expression was analyzed by a microarray using an antibody. Antibody microarray assay (Ray L1000 Antibody Array) can analyze 1000 genes and has 15 domains including inflammatory response, neurogenesis, RNA splicing, secretion, extracellular matrix, angiogenesis, cell cycle, apoptotic processes, cell differentiation, cell death, cell migration, cell proliferation, DNA repair, aging, and immune response. As a result of the analysis, in LmnaDhe/+ mutant MSCs including inflammatory response (14.94%), neurogenesis (12.08%), RNA splicing (0%), secretion (12.33%), aging (9.76%), angiogenesis (17.11%), apoptosis (11.90%), cell cycle (11.63%), cell death (10.95%), cell differentiation (11.46%), cell migration (13.76%), cell proliferation (11.76%), DNA repair (0%), extracellular matrix (13.27%) and immune response (13.25%) showed significant changes compared with normal MSCs (Figure 1A). An important point of these significant gene expression differences is that many of the significant gene expression differences were found to have reduced gene expression compared to normal MSCs (Figure 1B). By paying attention to genes involved in bone differentiation, it was confirmed that BMP-2 and osteocalcin, which are genes related to bone differentiation, all decreased compared to normal MSCs (Figure 1C).

### Osteogenesis of *Lmna* dysfunction MSCs compared with normal MSCs

**Figure 2:**
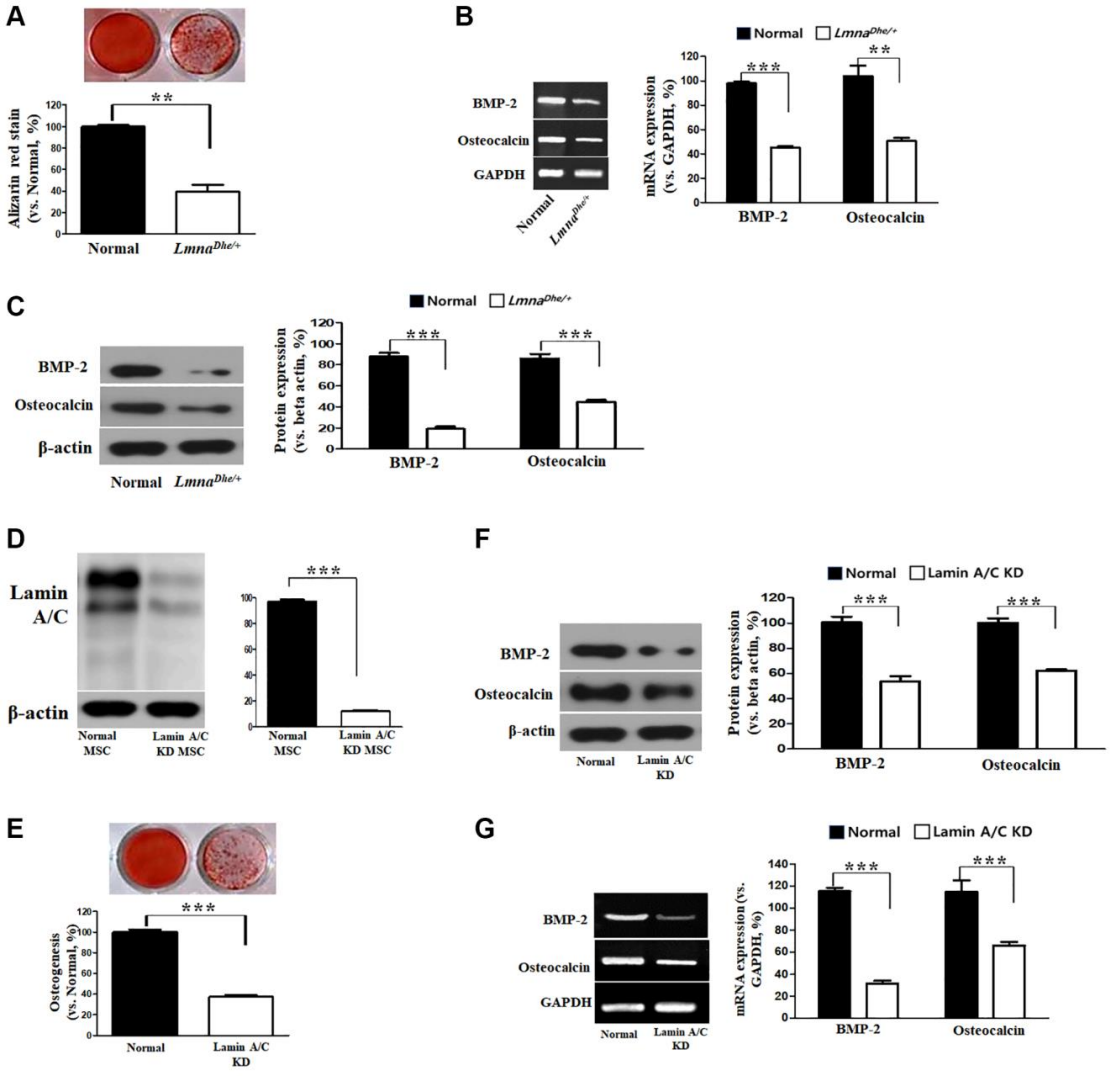
**Osteogenic analysis in *Lmna* dysfunction MSCs compared to normal MSCs.** (**A**) Alizarin Red staining. (**B**) Expression of osteogenic genes in *Lmna* dysfunction MSCs compared with normal MSCs. (**C**) Expression of osteogenic proteins in *Lmna* dysfunction MSCs compared with normal MSCs. (**D**) Lamin A/C knock down by siRNA. and (**E**) Alizarin Red staining. (**F**) Expression of osteogenic proteins in *Lmna* dysfunction MSCs compared with normal MSCs. (**G**) Expression of osteogenic genes in *Lmna* dysfunction MSCs compared with normal MSCs. ^***^p < 0.001 indicates a significant difference from the normal MSCs.

In this study, the osteogenic differentiation ability of *Lmna^Dhe/+^* mutant MSCs was confirmed compared to normal MSCs. After 3 weeks of induction of bone differentiation, Alizarin Red S staining was performed to confirm that bone differentiation of *Lmna^Dhe/+^* mutant MSCs was significantly reduced compared to that of normal MSCs (Figure 2A). Also, because of confirming the significantly decreased gene expression of BMP-2 and osteocalcin in the micro-array, it was also confirmed that the gene expression was significantly suppressed (Figure 2B). As a result of confirming the expression of osteogenic differentiation-related proteins including BMP-2 and osteocalcin, they all significantly decreased compared to normal MSCs (Figure 2C). Lamin A/C was knocked down by siRNA in MSCs (Figure 2D). It was confirmed compared to normal MSCs. As a result of confirming the expression of osteogenic differentiation-related proteins and genes including BMP-2 and osteocalcin, they all significantly decreased compared to normal MSCs (Figure 2E–2G).

### Pattern of gangliosides expression in *Lmna^Dhe/+^* mutant MSCs and Lamin A/C knockdown MSCs by siRNA

**Figure 3:**
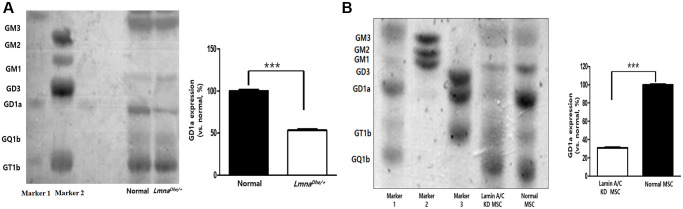
**Ganglioside expression patterns in *Lmna*-dysfunction MSCs.** (**A**) Ganglioside expression patterns in primary *Lmna^Dhe/+^* mutation MSCs. (**B**) Lamin A/C-knockdown MSCs by siRNA. ^***^p < 0.001 indicates a significant difference from the normal MSCs.

Gangliosides are known to affect cell proliferation and cell differentiation, and we were curious to know how these gangliosides affect *Lmna^Dhe/+^* mutant MSCs by HPTLC. As a result of the study, GM3, GM2, GM1, GD3, GQ1b, and GT1b were expressed similarly to normal MSCs, but ganglioside GD1a was significantly decreased compared to normal MSCs (Figure 3A). In addition, to further study the effect on the osteogenic differentiation of *Lmna* function-lose MSCs, Lamin A/C was knockdown (KD) by siRNA to confirm the expression of ganglioside. As a result, like *Lmna^Dhe/+^* mutant MSC, it was confirmed that only ganglioside GD1a was significantly decreased compared to normal MSCs (Figure 3B).

### Effects of ganglioside GD1a in osteogenesis of *Lmna^Dhe/+^* mutant MSCs and Lamin A/C knockdown MSCs

**Figure 4:**
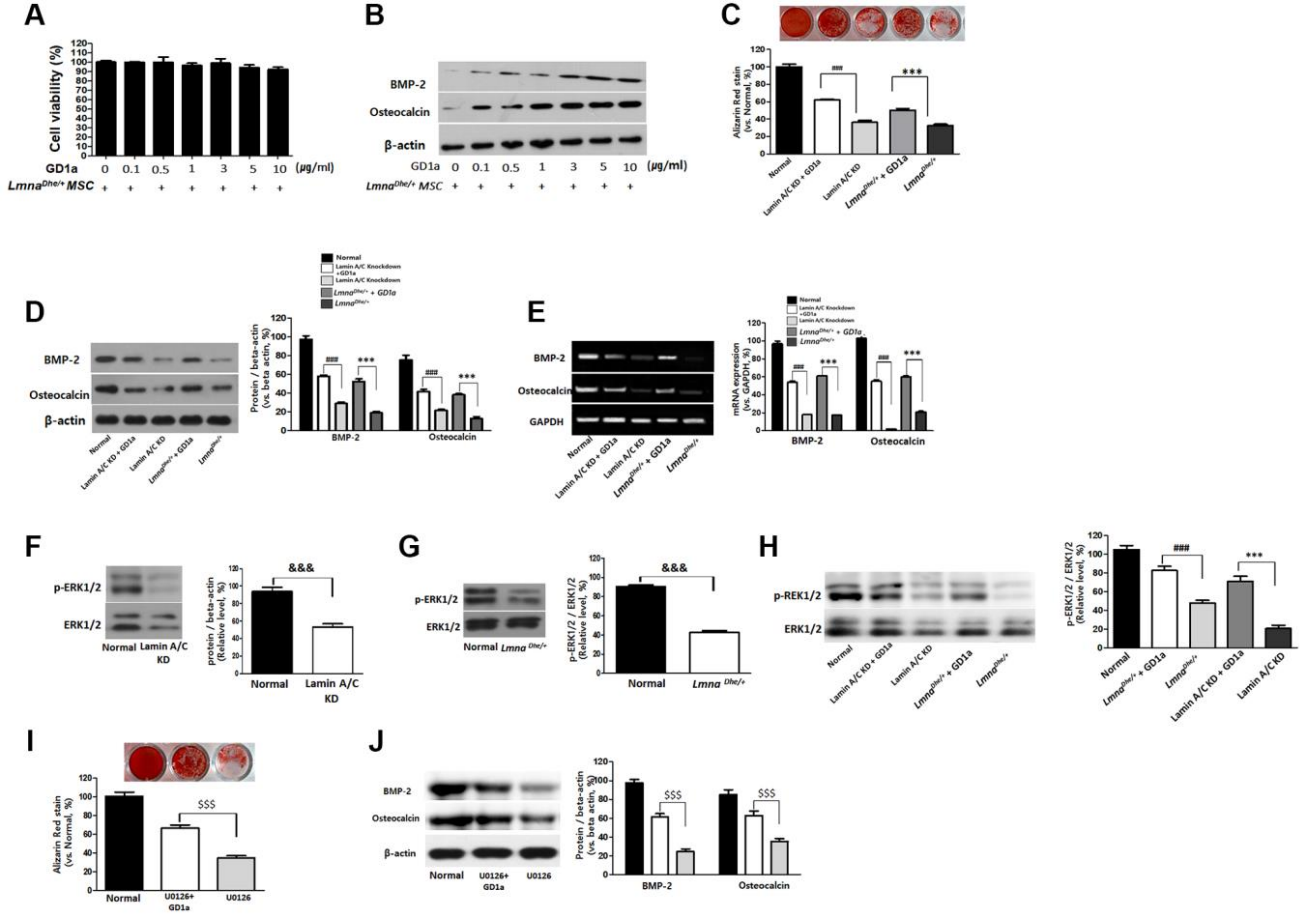
**Increases of osteogenesis and ERK1/2 activation in GD1a was-treated *Lmna* dysfunction MSCs.** Cell viability (**A**) and osteogenesis (**B**) in *Lmna^Dhe/+^* mutation MSCs were treated with GD1a. (**C**) Alizarin Red staining in GD1a was-treated *Lmna* dysfunction MSCs. (**D**) Increases of osteogenic proteins and (**E**) osteogenic genes in GD1a was-treated *Lmna* dysfunction MSCs compared with *Lmna* dysfunction MSCs. (**F**) Primary *Lmna^Dhe/+^* mutation MSCs were isolated from *Lmna^Dhe/+^* mutation mouse. (**G**) Lamin A/C was knocked down in mouse MSCs using siRNA. (**H**) Phosphorylation of ERK1/2 in *Lmna* dysfunction MSCs was treated with GD1a. (**I**) Alizarin Red staining in U0126-treated MSCs. (**J**) Increases of osteogenic proteins in GD1a-treated MSCs compared with pERK1/2-inhibited MSCs by U0126. Phosphorylation of ERK1/2 was determined by western blotting with anti-p-ERK1/2. ERK1/2 was used as a loading control. Values represent mean ± SD; ^&&&^*p* < 0.001 indicates a significant difference from the normal MSCs; ^***^p < 0.001 indicates a significant difference from the *Lmna^Dhe/+^* mutant MSCs; ^###^*p* < 0.001 indicates a significant difference from the Lamin A/C KD MSCs. ^$$$^*p* < 0.001 indicates a significant difference from the U0126-treated MSCs.

In this study, treatment of ganglioside GD1a with 0, 0.1, 0.5, 1, 3, 5, and 10 μg/ml was not cytotoxic (Figure 4A), and osteogenesis was significantly increased at 3, 5, and 10 μg/ml (Figure 4B). Therefore, we confirmed the osteogenic differentiation ability in ganglioside GD1a treated (3 μg/ml) *Lmna*^Dhe/+^ mutant MSCs after 3 weeks of osteogenic differentiation induction, it was confirmed through Alizarin Red staining. The bone differentiation of *Lmna*^Dhe/+^ mutant MSCs treated with ganglioside GD1a was significantly increased compared to that of *Lmna*^Dhe/+^ mutant MSCs (Figure 4C). In addition, as a result of confirming the expression of osteogenic differentiation-related proteins including BMP-2 and osteocalcin, they all significantly increased in *Lmna*^Dhe/+^ mutant MSCs treated with ganglioside GD1a (Figure 4D). It was confirmed that the gene expression of BMP-2 and osteocalcin, which were significantly decreased in the microarray, also increased significantly in ganglioside GD1a treatment MSCs (Figure 4E). Even in Lamin A/C knockdown MSCs, as a result of treatment with ganglioside GD1a, osteogenic differentiation was increased compared to Lamin A/C knockdown MSCs. In addition, the expression of osteogenic differentiation-related proteins such as BMP-2 and osteocalcin and an increase in gene expression was confirmed (Figure 4).

### Ganglioside GD1a increases the extracellular signal-regulated kinase 1/2 (ERK1/2) activation in osteogenesis of *Lmna^Dhe/+^* mutant MSCs and Lamin A/C knockdown MSCs

The activity of ERK1/2 activates osteoblast differentiation and bone formation. Therefore, we also confirmed the activity of ERK1/2 in MSCs with Lamin A loss of function. As a result, ERK 1/2 activity was significantly decreased in *Lmna*^Dhe/+^ mutant MSCs and Lamin A/C KD MSC than in normal MSC (Figure 4F, 4G). However, increased significantly in *Lmna*^Dhe/+^ mutant MSCs and Lamin A/C KD MSCs treated with ganglioside GD1a (Figure 4G). MSCs were preincubated with pERK inhibitor (U0126) at concentrations of 5 μM for 2 hr and then incubated in an osteogenic differentiation medium with the respective inhibitor for 21 days. Osteogenic differentiation of MSCs treated with pERK inhibitor (U0126) was significantly decreased compared to control MSCs (Figure 4I and 4J). However, in the MSCs treated with ganglioside GD1a, osteogenic differentiation was increased compared to MSCs treated with pERK inhibitor (U0126) (Figure 4I and 4J).

### Micro-array analysis of the ganglioside GD1a effect on gene expression in *Lmna^Dhe/+^* mutant MSCs

**Figure 5:**
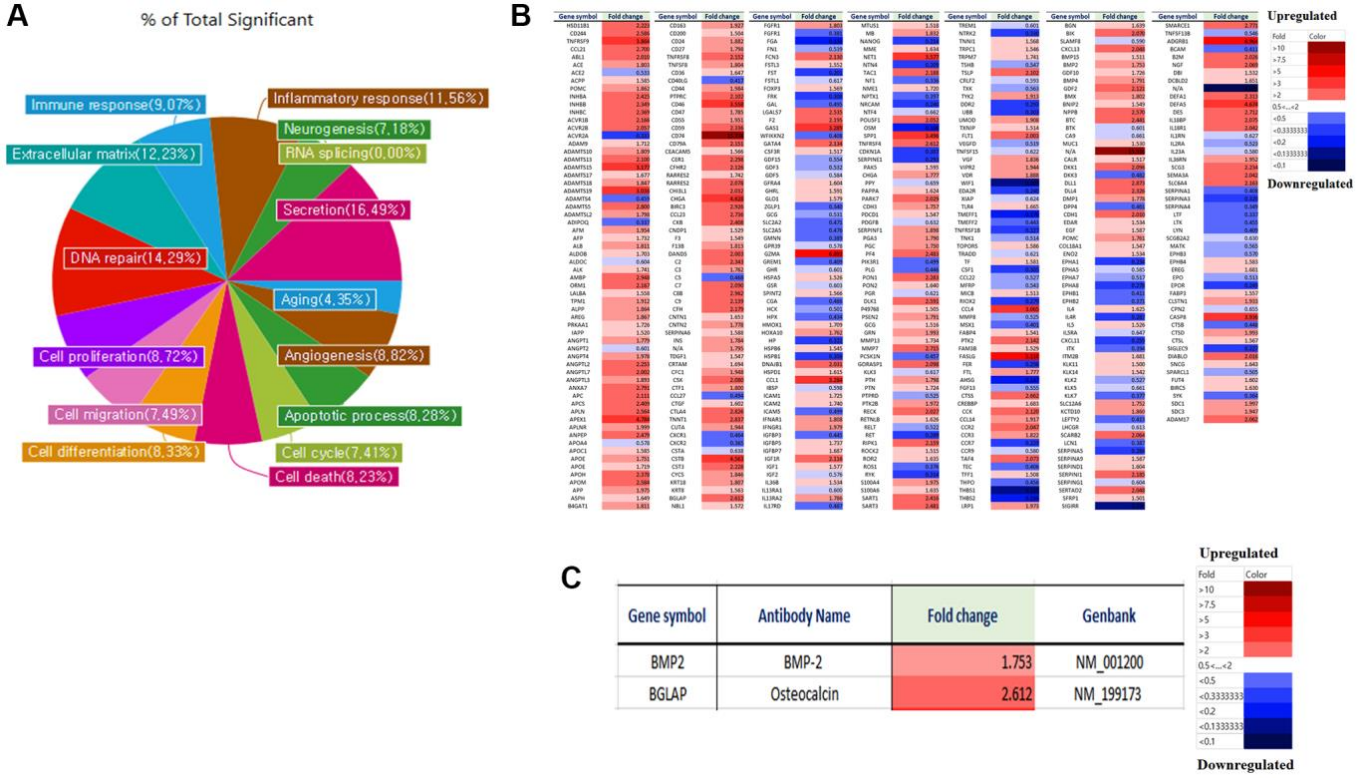
**Micro-array analysis of genes expression in *Lmna^Dhe/+^* mutant MSCs was treated with GD1a compared to *Lmna^Dhe/+^* mutant MSCs.** Gene expression was analyzed by a microarray using an antibody. (**A**) Antibody microarray assay (Ray L1000 Antibody Array) can analyze 1000 genes and has 15 domains such as inflammatory response, neurogenesis, RNA splicing, secretion, extracellular matrix, angiogenesis, cell cycle, apoptotic processes, cell differentiation, cell death, cell migration, cell proliferation, DNA repair, aging and immune response. (**B**) We analyzed the differences in gene expression in *Lmna^Dhe/+^* mutant MSCs was treated with GD1a compared to *Lmna^Dhe/+^* mutant MSCs. (**C**) Decreases of osteogenesis-related genes expression in *Lmna^Dhe/+^* mutant MSCs was treated with GD1a compared to *Lmna^Dhe/+^* mutant MSCs.

We analyzed the differences in gene expression in *Lmna^Dhe/+^* mutant MSCs with treated ganglioside GD1a compared to *Lmna^Dhe/+^* mutant MSCs. Gene expression was analyzed by a microarray using an antibody. Antibody microarray assay (Ray L1000 Antibody Array) can analyze 1000 genes and has 15 domains. As a result of the analysis, the genes expression in *Lmna^Dhe/+^* mutant MSCs with treated ganglioside GD1a including, inflammatory response (11.56%), neurogenesis (7.18%), RNA splicing (0%), secretion (16.49%), aging (4.35%), angiogenesis (8.82%), apoptosis (8.28%), cell cycle (7.41%), cell death (8.23%), cell differentiation (8.33%), cell migration (7.49%), cell proliferation (8.72%), DNA repair (14.29%), extracellular matrix (12.23%) and immune response (9.07%) showed significant changes compared to *Lmna^Dhe/+^* mutant MSCs (Figure 5A). An important point of these significant gene expression differences is that many of the significant gene expression differences were found to have significantly increased gene expression compared to *Lmna^Dhe/+^* mutant MSCs (Figure 5B). By paying attention to genes involved in bone differentiation, it was confirmed that BMP-2 and Osteocalcin, which are genes related to bone differentiation, all significantly increased compared to *Lmna^Dhe/+^* mutant MSC (Figure 5C).

### Effects of ganglioside GD1a in osteogenesis of *Lmna^Dhe/+^* mutant mouse

**Figure 6:**
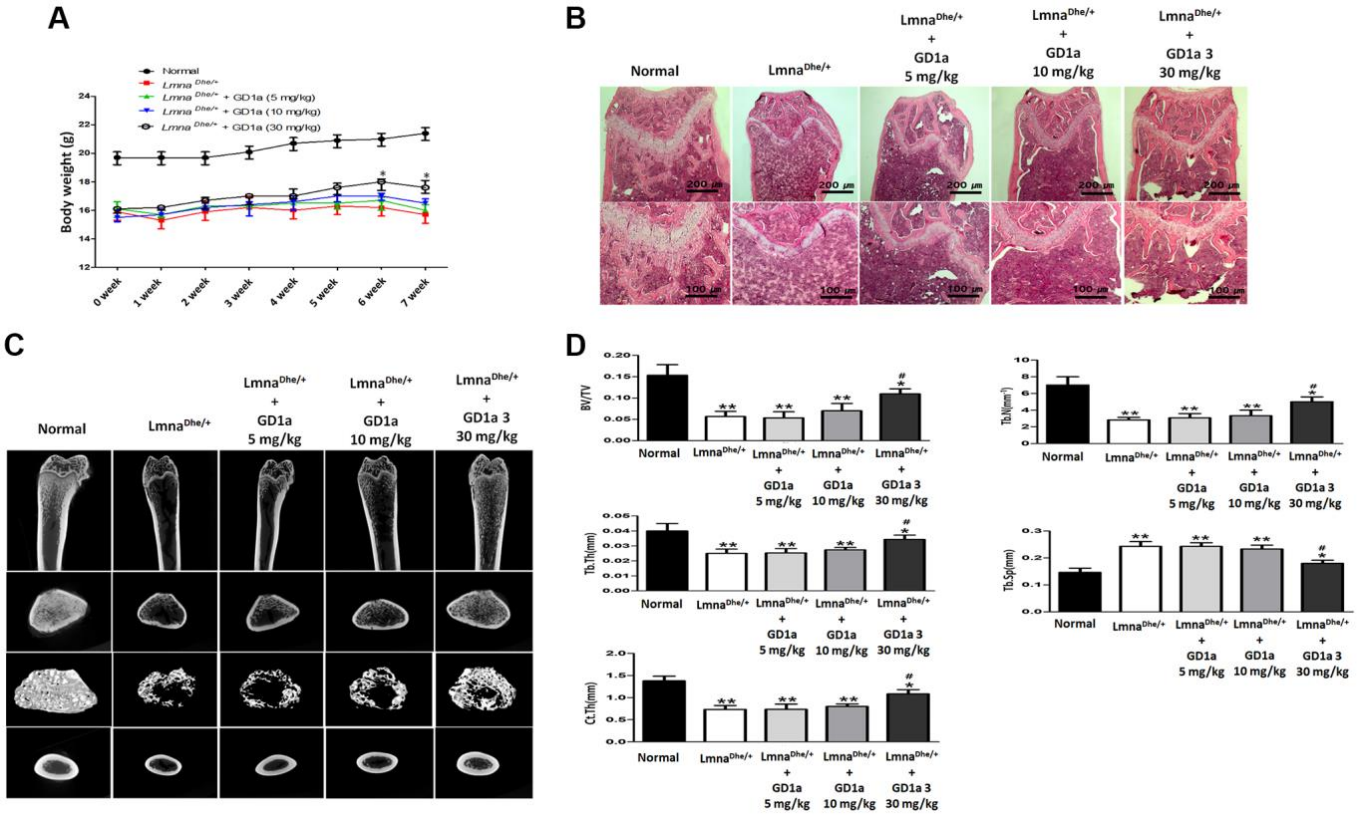
**Analysis of trabecular and cortical bone of the femur.*** Lmna^Dhe/+^* mutant mice were intraperitoneally injected with ganglioside GD1a at 5, 10, and 30 mg/kg/2-day for 7 weeks. (**A**) The mice were weighed once a week for 7 weeks, and (**B**) Hematoxylin and eosin staining of the femur nearby the metaphysic area. (**C**) Micro-CT analysis of trabecular and cortical bone of femur. (**D**) Representative parameters of trabecular and cortical bone, respectively. ^*^p < 0.05 indicates a significant difference the normal; ^**^p < 0.01 indicates a significant difference the Normal; ^#^*p* < 0.05 indicates a significant difference the *Lmna^Dhe/+^* mutant mouse.

To confirm the effect of ganglioside GD1a on bone formation in *Lmna^Dhe/+^* mutant mice GD1a was intraperitoneally injected at concentrations of 5, 10, and 30 mg/kg every 2 days for 7 weeks. As a result, the weight of GD1a-treated (30 mg/kg) mice showed a tendency to increase compared to the *Lmna^Dhe/+^* mutant mice at 6 weeks and 7 weeks compared to *Lmna^Dhe/+^* mice (Figure 6A). After 7 weeks, a histological examination showed bone volume differences in the femurs of *Lmna^Dhe/+^* mutant mice. Hematoxylin and eosin (H&E) stained sections showed a significant decrease in trabecular bone mass near the femoral growth plate. In addition, the interosseous cortical bone was thinner than that of normal mice. However, in *Lmna^Dhe/+^* mutant mice treated with ganglioside GD1a, trabecular bone and cortical bone were significantly increased than in *Lmna^Dhe/+^* mutant mice (Figure 6B). To determine the effect of GD1a on bone growth retardation and severe osteopenia in *Lmna^Dhe/+^* mutant mice, we performed bone histomorphometry using μCT analysis. Compared to normal mice, trabecular bone mass formation in *Lmna^Dhe/+^* mutant mice were significantly impaired with a decrease in trabecular bone volume and a decrease in the number of trabeculae per tissue volume, and trabecular space was increased simultaneously (Figure 6C, 6D). However, in the *Lmna^Dhe/+^* mutant mice treated with GD1a (30 mg/kg), the bone volume and the number of trabeculae per tissue significantly increased, and the trabecular space was significantly decreased compared to the *Lmna^Dhe/+^* mutant mice (Figure 6C, 6D). Moreover, the cortical bone thickness of *Lmna^Dhe/+^* mutant mice was significantly decreased compared to normal mice. However, the cortical bone thickness of GD1a-treated (30 mg/kg) *Lmna^Dhe/+^* mutant mice was significantly increased compared to *Lmna^Dhe/+^* mutant mice, as observed in the longitudinal and transverse mid-diaphysis sections (Figure 6C, 6D).

## DISCUSSION

Lamin A/C is a structural protein encoded by the *Lmna* gene. *Lmna* is an essential scaffolding component of the nuclear membrane that surrounds the cell nucleus. The Dhe mutation is the first spontaneous mutation of the *Lmna* gene in mice. Several studies to date have focused on abnormalities of the skull, bones, skin and fat, and the nuclei of cultured connective tissue cells. We hypothesize that ganglioside has an important relationship in the *Lmna* mutation model (progeria), but the distribution and role of ganglioside in the *Lmna* mutation model remain unclear. Bone formation by *Lmna* mutation appears to be affected, but sufficient studies have not been conducted to confirm this with certainty so far. Therefore, in this study, the association of gangliosides in bone loss caused by *Lmna* mutation was confirmed.

The *Lamin* protein family, of which Lamin A/C is a member, plays important roles in nuclear stability, chromatin structure, and gene expression [1]. Therefore, the genetic changes of MSCs in *Lmna^Dhe/+^* mice and MSCs in normal mice were investigated. As a result, inflammatory responses, neurogenesis, RNA splicing, secretion, senescence, angiogenesis, apoptotic processes, cell cycle, apoptosis, cell migration, cell differentiation, DNA repair, cell proliferation, extracellular matrix, and immune responses were significantly reduced in *Lmna* mutation (Figure 1). These results demonstrate that *Lmna* gene mutations are responsible for bone dysplasia, adipogenesis, and progeria. It was demonstrated that the gene reduction of BMP-2 and osteocalcin, which are osteogenic-related genes, affected the osteogenic differentiation capacity of *Lmna* mutant mesenchymal stem cells, thereby causing abnormal bone formation.

MSCs are precursors to osteoblasts, the cells that form bones. MSCs undergo a multistep process of bone formation mainly in response to various signals from the surrounding ECM [14, 15]. Moreover, during bone healing, inflammatory mediators activate and mobilize tissue resident endogenous MSCs that migrate from the niche to the damaged area to promote bone tissue regeneration [16]. To achieve both migration and osteogenic differentiation, MSCs must reorganize their nuclear layer morphology or organization. Therefore, it is known that the level of Lamin A increases during osteogenesis of MSCs and decreases during adipogenesis [17, 18]. Similarly, conditions that affect Lamin A/C, such as aging and LMNA mutations, negatively affect MSCs migration and osteogenesis and affect bone tissue homeostasis. Osteogenesis can be measured *in vitro* and *in vivo* by various markers such as osteoactivin, osteocalcin, alkaline phosphatase expression, and calcium deposition. The effects of Lamin A/C on bone formation have been evaluated *in vitro* in several studies. A significant decrease in Alizarin red staining was observed in Lamin A/C inhibited MSC and mature osteoblasts, confirming a significant decrease in osteoblast differentiation and mineralization. In addition, significantly lower levels of osteoactivin and osteocalcin were measured in *Lmna* knockdown cells [19]. In this study, as a result of confirming the osteogenic differentiation capacity of *Lmna^Dhe/+^* mutant MSCs, it was confirmed that the expression of BMP-2 and osteocalcin proteins and genes, which are important factors for osteogenic differentiation, were all reduced. As shown in Figures 3, it was confirmed that BMP-2 and osteocalcin protein and gene expression were all decreased to suppress osteogenic differentiation. These results suggest that the inhibition of osteogenic differentiation was induced by loss of function of the *Lmna* gene.

Gangliosides are also known to be involved in key processes in cell proliferation and differentiation [20, 21]. In this study, we observed the gene expression patterns of *Lmna^Dhe/+^* mutant MSCs significantly decreased compared to normal MSCs (Figure 1). Also, ganglioside GD1a in *Lmna^Dhe/+^* mutant MSCs significantly decreased compared to normal MSCs (Figure 3). However, treatment of ganglioside GD1a in *Lmna^Dhe/+^* mutant MSCs increased gene expression patterns compared to *Lmna^Dhe/+^* mutant MSCs (Figure 5). BMP-2 and osteocalcin, which were significantly reduced in *Lmna^Dhe/+^* mutant MSCs (Figure 2), were significantly increased by ganglioside GD1a (Figure 4). This suggests that GD1a plays an important role in osteogenic gene expression. Previous reports have shown that ganglioside GD1a is increased during osteogenic and neuronal differentiation, suggesting a possible role for this ganglioside in directing this process [22, 23]. Gangliosides during the differentiation of human MSCs (hMSCs) to osteoblasts are known to functionally modulate growth factor receptors [24]. In a previous study, have reported that only the gangliosides GM3, GM2, and GD1a influenced osteogenic formation [11]. Also, in previous studies have reported that the ganglioside GD1a was significantly increased during MSC differentiation into osteoblasts [11, 25]. Inhibition of ganglioside GD1a synthesis by knockdown of ST3Gal II mRNA, a restriction enzyme for ganglioside GD1a synthesis, may interfere with osteoblast differentiation of hMSCs [26]. Thus, those reports suggest that the ganglioside GD1a plays an important role in regulating the differentiation of MSCs into osteoblasts. These findings support the result that the reduction of ganglioside GD1a in the *Lmna* gene mutation in this study results in inhibition of MSCs osteo-differentiation. Furthermore, we suggest that ganglioside GD1a plays an important role in the enhancement of osteogenic differentiation of *Lmna* mutant MSCs and femur formation in *Lmna^Dhe/+^* mutant mice.

Several studies have reported that ERK1/2 is involved in the regulation of osteoblast differentiation [27, 28]. The canonical ERK has two isoforms, ERK1and ERK2, both of which are highly expressed in cells of the osteoblast lineage. Therefore, in this study, it was confirmed that ERK1/2 activity was decreased in *Lmna* mutant MSCs (Figure 4F, 4G), and ERK1/2 activity was increased as a result of ganglioside GD1a treatment (Figure 4H). These results suggest that the bone loss of the *Lmna* mutant is due to the inhibition of ERK1/2 activity and that ganglioside GD1a enhances the *Lmna* mutant bone formation by increasing the activity of ERK1/2.

It was confirmed that the expression of various genes, including ontogenetic differentiation-related genes, was decreased in *Lmna^Dhe/+^* mutant MSCs compared to normal MSCs. Osteogenic differentiation of *Lmna* mutant MSCs was significantly lower than that of normal MSCs, and related gene expression and protein expression were also lower. In particular, the ganglioside GD1a was significantly reduced in *Lmna^Dhe/+^* mutant MSCs with low osteogenic differentiation. These results suggest that ganglioside GD1a plays an important role in osteogenic differentiation due to *Lmna* dysfunction. In addition, as a result of confirming that *Lmna^Dhe/+^* mutant mice were treated with ganglioside GD1a, body weight and femur bone volume (osteogenesis) were improved compared to *Lmna^Dhe/+^* mutant mice (Figure 6). Therefore, these results suggest that ganglioside GD1a plays an important role in the enhancement of bone formation in *Lmna^Dhe/+^* mutants.

## MATERIALS AND METHODS

### Mice

*Lmna^Dhe/+^* heterozygous mice were obtained from Jackson Laboratory (Bar Harbor, ME, USA). The strain was maintained heterozygous as homozygous *Lmna^Dhe/Dhe^* pups die at approximately P10. All animal experiments were performed according to standard operating procedures and were approved by the Animal Care Committee of Wonkwang University, Korea (approval number WKU18-35).

### Primary culture

Primary cultures of mouse BM-MSCs were arranged by modifying the method of Li et al. (Li et al., 2016). BM-MSCs plated at a density of 160,000/cm^2^ in complete culture medium in 75–150 cm^2^ tissue culture flasks (BD Falcon, NJ): DMEM supplemented with 10% fetal bovine serum (FBS) (Gibco, Life Technologies Ltd, Paisley, UK) L-Glutamine (2 mM) (L-Glu, Euroclone), Streptomycin (50 mg/ml) and Penicillin (50 U/ml). After culturing for 48 hours, the culture medium was replaced at 3 days. BM-MSCs were reaped after attainment ≥70% confluence using Trypsin (Euroclone) and propagated to 4,000 cells/cm^2^ until attainment replicative senescence.

### Immunophenotype by FACS

MSCs were phenotypically considered by flow cytometry at P3-P4 to assess the presence of the surface markers CD73, CD90 and the absence of CD34, CD45 and fluorescein isothiocyanate (FITC) or phycoerythrin (PE)-conjugated monoclonal antibodies (all from BD PharMingen, San Diego, CA, USA). Analysis of cell populations was achieved by direct immunofluorescence using a FACS flow cytometer (BD PharMingen). Calculations were performed with FACS Diva software (Tree Star, Inc. Ashland, OR, USA).

### Antibody microarray analysis

Antibody microarray analysis (Ray L1000 Antibody Array) was commissioned by ebiogen (Gyeonggi-do, Korea). The Ray L1000 Antibody Array can confirm the expression of 1,000 genes in *Lmna^Dhe/+^* mutant MSCs compared with normal MSCs. Since it is a linear scale, starting from 1, if the value is less than 1, expression decreases than control, and if it is greater than 1, expression increases. This is the value converted to log^2^ value after global normalization of raw data.

### Western blot

MSCs were lysed with cold Pro-PREPTM buffer (INtRON Biotechnology, Gyeonggi-do, Korea). MSCs were centrifuged at 14,000 rpm for 20 min at 4°C. Protein concentrations were determined using the BCA Protein Assay Kit (Pierce, Rockford, IL, USA). Protein extracts were separated on 10% SDS polyacrylamide gels and transferred to poly (vinylidene fluoride) membranes at 200 mA for 1 h. Membranes were blocked with PBS containing 5% bovine serum albumin for 1 h at room temperature and then incubated with primary antibodies including anti- extracellular signal-regulated kinase 1/2, anti-bone morphogenetic protein-2, anti-β-actin and anti-osteocalcin (1:1000 dilution, Cell Signaling, Beverly, MA, USA), overnight at 4°C, followed by horseradish peroxidase-conjugated Anti-rabbit secondary antibody (1:1000 dilution, Cell Signaling) for 1 h at room temperature. Peroxidase activity was estimated using an enhanced chemiluminescence (ECL) kit (Thermo Fisher Scientific, Waltham, MA, USA).

### High performance thin-layer chromatography

High-performance thin-layer chromatography (HPTLC) analysis of the gangliosides was directed using a 10 × 10 cm TLC 5651 plate (Merck, Darmstadt, Germany). The purified gangliosides (600 μg protein/lane) were loaded onto TLC 5651 plates that were after developed in chloroform/methanol/0.25% CaCl_2_·H_2_O (50:40:10, v/v/v). The gangliosides are contained with resorcinol, after which the densities of the ganglioside bands were quantified by HPTLC densitometry (Image J, NIH). Purified mixed gangliosides GM3, GM2, GM1, GD3, GD1a, and GD1b (Matreya LLC, Pleasant Gap, PA, USA) were used as standards.

### Design and selection of allele-specific siRNA

Lamin A/C synthase-specific siRNA and control siRNA were synthesized by Bioneer Inc. (Daejeon, Korea). The primers for Lamin A/C synthetase are F, 5′-GAGAUCGAUAACGGGAAGC-3′ and R, 5′-GCUUCCCGUUAUCGAUGUC-3′. All sequences were confirmed by capillary sequencing. Transfection of siRNA was performed using Lipofectamine 3000 reagent (Invitrogen, Carlsbad, CA, USA) according to the manufacturer’s recommendations. Lamin A/C synthase specificity of siRNA was determined using western blot by comparing the activity of Lamin A/C.

### Osteoblastic differentiation assays

Assays were achieved on cultured MSCs at passage 2–3. Cells were stimulated with differentiation medium for 3–4 weeks, and the medium was changed every 3 days. For osteogenic differentiation, cells were treated with 10% FBS, 10 nM HEPES, 100 unit/ml penicillin, 100 μg/ml streptomycin, 50 μg/ml ascorbic acid (Sigma-Aldrich), 5 × 10^−7^ M dexamethasone (Sigma-Aldrich) and 10 mM glycerol phosphate. Cells were fixed with 10% formalin, and calcium deposition was confirmed using 1% alizarin red S (Sigma-Aldrich) (pH 4.1). Alizarin red (sodium alizarin sulfonate) staining, 1% alizarin red was ready in distilled water, and the pH was adjusted to 4.1 using 0.5% ammonium hydroxide. Cultures were stained with alizarin red S for 15 min after fixation. After removal of the unmixed excess dye with distilled water, the mineralized nodules appeared as red spots [26]. After Alizarin red s staining, 10% cetylpyridinium chloride was added to 10 mM sodium phosphate (pH 7.0) and dissolved for 15 minutes, and the degree of staining was measured for absorbance at 562 nM.

### RT-PCR

The cDNA for RT-PCR was obtained by PCR amplification using primers specific to GAPDH, bone morphogenetic protein-2 (BMP-2) and osteocalcin. RT-PCR was performed using TaKaRa Ex Taq (Takara, Dalian, China) with the TaKaRa PCR Thermal Cycler Dice Gradient system (Takara, Dalian, China).

### Inhibition of the MAPK signaling pathways by inhibitor (U0126)

BMSCs were preincubated with pERK inhibitor (U0126) at concentrations of 5 μM for 2 hr and then incubated in osteogenic differentiation medium with the respective inhibitor for 21 days. Concentration of U0126 inhibitor was chosen based on previous study (Doan et al., 2012).

### Ganglioside GD1a treatment *in vitro* and *in vivo*

Ganglioside GD1a was purchased from Sigma-Aldrich (St. Louis, MO, USA). To check the effect of ganglioside GD1a on bone differentiation, it was treated in MSCs with loss of Lamin A function at concentrations of 0.1, 0.5, 1, 3, 5, and 10 μg/ml. Antibody microarray analysis was performed to confirm the effect of ganglioside GD1a on gene expression patterns due to loss of Lamin A function. MSC medium was replaced every 3 days and ganglioside GD1a was treated every 3 days for 21 days. *Lmna^Dhe/+^* mutant mice were intraperitoneally injected every 2 days for 7 weeks with ganglioside GD1a at concentrations of 5, 10, and 30 mg/kg. The mice were weighed once a week for 7 weeks, and the bone volume of the femur was measured at an autopsy after 7 weeks.

### Bone histomorphometry

For histological analysis, the femurs were fixed with 10% phosphate-buffered formalin and decalcified, embedded in paraffin, and stained with hematoxylin and eosin. The bone microstructure of the distal femur samples was evaluated by μCT (Skyscan 1176; Skyscan, Antwerp, Belgium) and related software (CT-analyser, version: 1.10.11.0). For the femoral neck, VOIs (volumes of interest) of the same size were sampled from the image stack. Each VOI in the femoral neck trabecular was oriented parallel to the axis of the femoral neck, centered on the femoral neck around the narrowest region, approximately 2 mm distal to the femoral head growth plate. Trabecular parameters for femurs include trabecular bone volume (BV), total bone volume (TV), fraction (BV/TV), number of trabecular (Tb.N), trabecular thickness (Tb.Th), and trabecular separation (Tb.Sp) calculated with Skyscan software CTscan. Cortical thickness (Ct.Th) was shown semi-automatically on the images by contouring using the same set of slices for trabecular bone measurements.

### Statistical analysis

All data can be accessed as mean (SD). Multiple group associations were run using one-way ANOVA and two-way ANOVA followed by Tukey and Bonferroni post hoc pairwise comparisons. *P* values < 0.05 were considered statistically significant. All statistical investigations were performed using GraphPad Prism (Ver. 5.00; GraphPad Software Inc., La Jolla, CA, USA).

## Supplementary Materials

Supplementary Figure 1
