# Correction for: Ganglioside GD1a enhances osteogenesis by activating ERK1/2 in mesenchymal stem cells of *Lmna* mutant mice

**DOI:** 10.18632/aging.204489

**Published:** 2023-01-14

**Authors:** Dong Hoon Kwak, Ji Hye Park, Eul Sig Choi, Seong Hyun Park, Seo-Yeon Lee, Seoul Lee

**Affiliations:** 1 Department of Pharmacology, School of Medicine, Wonkwang University, Iksan, Jeollabuk-do 54538, Republic of Korea; 2 Brain Research Institute, Wonkwang University, Iksan, Jeollabuk-do 54538, Republic of Korea

**Keywords:** Lmna, mesenchymal stem cells, osteogenesis, GD1a, aging

**This article has been corrected:** The authors found that Western blot images of p-ERK1/2 and ERK1/2 **in Figure 4F** were from different experiment with protein extracts from *Lmna^Dhe/+^*mice. They replaced the incorrect images with the images of p-ERK1/2 and ERK1/2 in MSC protein extracts from normal and Lamin A/C knockdown (KD) mice. In **Figure 4H**, the authors corrected a typo where “p-ERK1/2” was mislabeled “p-REK1/2”. The authors also revised the Figure legend for **Figure 4F** and **4G** as follows: “(**F**) Lamin A/C was knocked down in mouse MSCs using siRNA. (**G**) Primary MSCs with *Lmna^Dhe/+^* mutation were isolated from *Lmna^Dhe/+^* mice.” These corrections have no impact on the experimental outcome or conclusions.

Corrected **Figure 4** legend and panels **4F** and **4G** presented below.

**Figure 4:**
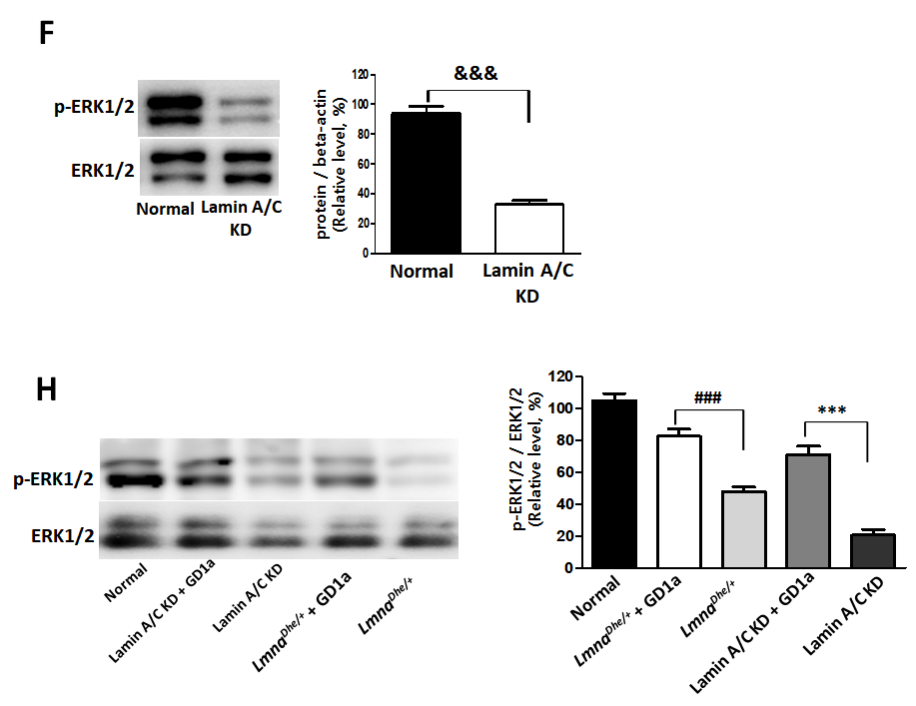
**Increases of osteogenesis and ERK1/2 activation in GD1a-treated *Lmna* dysfunction MSCs.** *…* dysfunction MSCs. (**F**) Lamin A/C was knocked down in mouse MSCs using siRNA. (**G**) Primary MSCs with *Lmna^Dhe/+^* mutation were isolated from *Lmna^Dhe/+^* mice. (**H**) Phosphorylation of ERK1/2 in *Lmna* dysfunction MSCs treated with GD1a. (**I**)…. Phosphorylation of ERK1/2 was determined by western blotting with anti-p-ERK1/2. ERK1/2 was used as a loading control. Values represent mean ± SD; ^&&&^*p* < 0.001 indicates a significant difference from the normal MSCs; ***p < 0.001 indicates a significant difference from the *Lmna^Dhe/+^* mutant MSCs; ^###^*p* < 0.001 indicates a significant difference from the Lamin A/C KD MSCs. ^$$$^*p* < 0.001 indicates a significant difference from the U0126-treated MSCs.

