# Supplementary material for: Ganglioside GD1a enhances osteogenesis by activating ERK1/2 in mesenchymal stem cells of Lmna mutant mice
Source: Aging (Albany NY). 2022 Nov 14;14(23):9445–57. doi: 10.18632/aging.204378 (PMC9792213; doi:10.18632/aging.204378)
Supplement: Supplementary Figure 1 [file aging-14-204378-s001.pdf]

SUPPLEMENTARY FIGURE

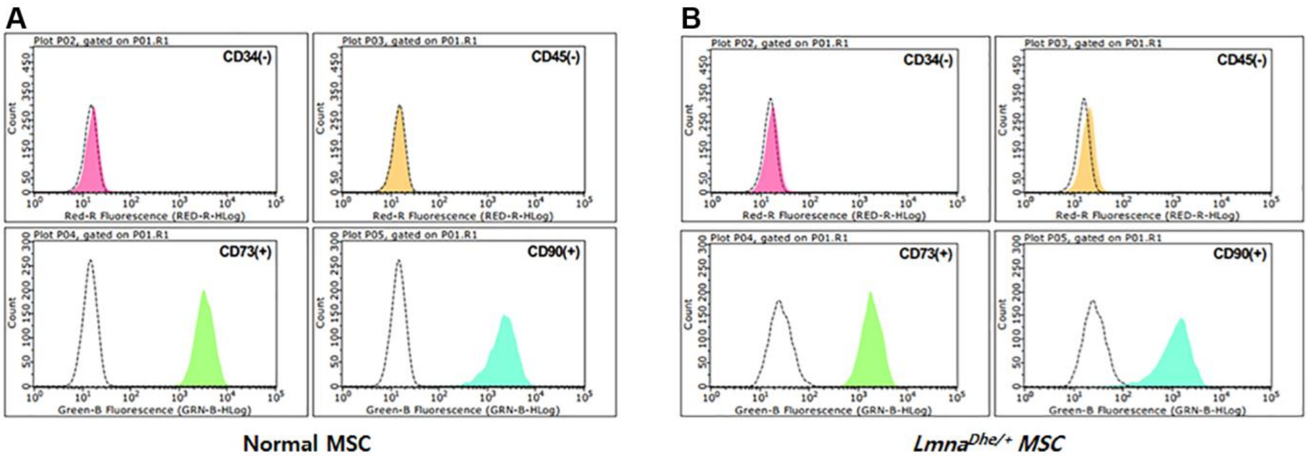

Supplementary Figure 1. Characterization of BMMSCs. Flow cytometric analysis of antigens CD34, CD45, CD73, and CD90. Negative control: CD34 and CD45; Positive control: CD73 and CD90. (**A**) Normal MSCs, (**B**) *Lmna*$^{Dhe/+}$ mutation MSCs.
